# The factors that affect the mortality of emergency operated ASA 3 colon cancer patients

**DOI:** 10.11604/pamj.2020.36.290.24385

**Published:** 2020-08-17

**Authors:** Yeliz Yilmaz, Fevzi Cengiz, Erdinç Kamer, Turan Acar, Emine Özlem Gür, Halis Bag, Yasin Peker, Kemal Atahan

**Affiliations:** 1 Department of General Surgery, Izmir Katip Celebi University, Ataturk Training and Research Hospital, Izmir, Turkey

**Keywords:** Colon cancer, ASA classification, emergency surgery, mortality

## Abstract

**Introduction:**

colorectal cancers take third place among cancer-related deaths and 10-28% of these patients are admitted with the necessity of emergency surgical intervention. The main propose of this study was to investigate the factors affecting mortality in ASA 3 colorectal cancer patients who undergo emergency surgery.

**Methods:**

between 2010 and 2017 ASA 3 patients who underwent emergency colon cancer surgery were included in the study. All of the study group was evaluated within the first 30-day time-frame. The results were obtained by a statistical comparison of the data of patients with and without mortality.

**Results:**

one hundred and twenty eight patients included in the study. There was no statistical difference in the demographic data of the groups and the indications of the operation. The differences and durations of surgery also did not make any statistical difference. The complication rate was the same according to the Clavien-Dindo classification.

**Conclusion:**

despite the screening programs applied in colorectal cancers, applications to emergency services and procedures performed under emergency conditions are still at high levels. Surgical operations, which have to be performed in patients with impaired metabolic status, carry major risks for patients, but their outcomes are also satisfactory for them.

## Introduction

Colorectal cancers take the third place among cancer-related deaths in men and women [1]. They are often seen in the elderly population. Its prevalence is 1.6% in the 50-60 age group and 3% in the 70-age group [1]. It may manifest itself with numerous signs and symptoms as abdominal pain, changes in stool, loss of appetite, diarrhea, constipation, fatigue, feces with mucus, nausea, vomiting, rectal pain-bleeding and tenesmus. While patients are admitted with the necessity of emergency surgical intervention because they ignore the relevant the symptoms [2]. In the surgical treatment of obstructive colorectal cancers under emergency conditions, higher rates of morbidity and mortality are observed when compared with treatment under elective conditions [3]. Metabolic, cardiovascular, infectious, or respiratory tract problems may occur in these patients and result in worse outcomes when compared to elective surgery [4]. In this case, there are many treatment options such as ostomy, resection + ostomy or resection + anastomosis. In the literature, there are articles suggesting that only ostomy should be opened, but there are also reports indicating that resection may be also performed [5]. In the evaluation of the preoperative medical condition, the five-grade patient classification proposed by the American Society of Anesthesiologists (ASA) is used and the established criteria are as follows: ASA 1, normal healthy patient; ASA 2, patient with mild systemic disease; ASA 3, patient with severe systemic disease which is not incapacitating; ASA 4, patient with incapacitating systemic disease which is a constant threat to life; and ASA 5, moribund patient not expected to live twenty-four hours [6]. The aim of the present study was to investigate the factors affecting mortality within one month after surgery in ASA 3 patients who were followed up for colorectal cancer and had to undergo emergency surgery.

## Methods

**Study design:** we retrospectively evaluated 128 ASA 3 colorectal cancer patients who were operated between January 2010 and January 2018 under emergency conditions in our clinic. Data were obtained from patient files, patient discharge summaries, surgical notes and observation papers in the hospital archive. All patients applied to the emergency department and following the necessary fluid and electrolyte treatment, appropriate consultations were made and they were immediately taken into operation. The type of surgery to be performed was chosen according to the location of the tumor, viability and dilatation of the intestines, general condition of the patient and the presence of comorbid disease. Operative mortality was used to describe deaths happening within 30 days after surgery. The patients were divided into two groups as those died (n = 40) or survived (n = 88) within 30 days after surgery.

**Outcome parameters:** demographic data, operative indications, type of surgery, operative times, operative complications and mortality were retrospectively reviewed from prospectively kept records in the hospital database. Complications were evaluated according to Clavien-Dindo classification. The results obtained were based on statistical comparison of the data of the exited and survived patients.

**Statistical analysis:** SPSS 15.0 for Windows was used to evaluate the results. The t-test and Mann-Whitney U-test were used to evaluate the independent groups, p<0.05 was considered statistically significant.

## Results

A total of 1434 patients with colorectal cancer were undergone operation in our department within the specified time period. A total of 128 of them (8.9%) were emergent cases. Of the 128 cases, 40 of them died within 30 days after surgery, while 88 survived. The patients were divided into two groups as those died (n = 40) or survived (n = 88) within 30 days after surgery. Of the 128 patients (80 males, 48 females) whose charts were reviewed, the mean age was 68.4 ± 14.6 (range 29 to 98) years. Mortality group included 40 patients (26 males, 14 females) with a mean age of 71.2 ± 14.5 years. Survival group included 88 patients (54 males, 34 females) with a mean age of 67.1 ± 14.6 years. Both groups did not differ from each other by means of age and gender (p = 0.154 and p = 0.056 respectively) (Table 1). In the mortality group, emergency surgical indications were acute mechanical intestinal obstruction in 30 patients (75%), perforation in 5 (12.5%) patients, anastomotic leak in 3 (7.5%) patients and fistula in 2 (5%) patients. In the survival group, surgical indications were obstruction in 57 patients (64.8%), perforation in 12 (13.6%) patients, anastomotic leak in 17 (19.3%) patients and fistula in 2 (2.3%) patients. No statistically significant difference was observed between the groups in terms of operative indications (p = 0.944). No statistically significant difference was observed between the groups in terms of type of surgery and duration of surgery (Table 1). Anastomotic leak was observed in two patients who underwent anastomosis after resection and who did not undergo ostomy. Mortality was not observed in these patients. There was no statistically significant difference between the two groups in terms of Clavien-Dindo classification (2%, 21.0%, 17.8%, p = 0.577).

**Table 1:** demographic data, indications, operative data and operative times of the patients

		Short-term Mortality Group (n=40)	Short-term Survival Group (n=88)	p-Value
Age (year)		71.2 ± 14.5	67.1 ± 14.6	0.154
Gender (male/female)		26/14	54/34	0.056
Primary/Secondary		35/5	70/18	
Indications of surgery	Obstruction	30/40 (75%)	57/88 (64.8%)	0.944
	Perforation	5/40 (12.5%)	12/88 (13.6%)	
	Anastomotic leak	3/40 (7.5%)	17/88 (19.3%)	
	Fistula	2/40 (5%)	2/88 (2.3%)	
Surgery	Ostomy	35/40 (87.5%)	62/88 (70.5%)	0.074
	Resection + Ostomy	5/40 (12.5%)	26/88 (29.5%)	
Operative time		124.4±23.7	116.7±23.3	0.246

## Discussion

This study was to evaluate the factors affecting the death of patients with ASA 3 colon cancer during 30 days after surgery performed under emergency conditions but no significant difference was observed between the patient groups. The average age of onset of colon cancer was 63 years in males and 62 years in females [7]. With age, both the incidence of the tumor and the increase in complications and mortality rates due to the surgical intervention are observed. There are different results related to the distribution of colorectal cancers according to gender in the literature [1, 7]. Although it has been reported that it has similar incidence rates in both sexes [8]. In our study, no statistically significant difference was found between the study groups in terms of age and male/female distribution. Leung and Dzankic reported higher ASA scores as the risk factors that could lead to the development of complications during and after the surgery [9]. In colon cancer patients, the frequency of accompanying comorbidities and high ASA score have been shown to increase postoperative morbidity and mortality as independent risk factors [10, 11]. Many of the complications emerging related to colon cancers are those requiring urgent intervention for patients. However, long-term results of the patients operated under emergency conditions are known to be worse than the elective patients in the same stage. Therefore, if possible, we think it would be appropriate to convert the surgical intervention into the elective approach.

The most common complication in colon cancer patients is obstruction, followed by perforation. In the present study, 67.9% of the whole study group had obstruction and 13.3% of patients had perforation. Acute intestinal obstruction occurs in 8-29% of patients with colorectal cancer. These patients have locally advanced disease or metastases that often make resection impossible [12]. The two most common approaches to these patients are colostomy and resection of the primary tumor if appropriate. Most patients are comorbid, malnourished, dehydrated and elderly. Hartmann´s procedure is the best approach for the surgical resolution of the obstruction due to having a lower mortality rate compared to other options [13]. In the present study, emergency surgical indication was obstruction in 75% of patients in the mortality group, while 64.8% in the survival group. Perforation is an important complication which causes intraabdominal sepsis. Perforations are mostly seen in advanced stage tumors. Mortality rates in cases with tumor perforation reach up to 30-40% [10]. When short-term death rates after the intervention are excluded mortality rates in colorectal cancers associated with perforation are parallel to other colorectal cancers that were detected at the same stage [14]. In the present study, emergency surgical indication was perforation in 12.5% of patients in the mortality group, while 13.6% in the survival group. In general, it is recommended to perform stepwise surgery in colon tumors, because resection and primary anastomosis in an extremely dilated and dirty colon under emergency conditions is considered to be highly risky. Its biggest risk is anastomotic leak [15].

Although there are opposing views in different publications, the most frequently preferred emergency surgery for left colon cancer appears to be resection and Hartmann surgery. Seah *et al*. concluded that Hartmann's operation was a useful procedure in 85 cases of elderly patients with colorectal cancer including 45 cases with medical problems [16]. Ansaloni *et al*. emphasized that Hartmann surgery should be preferred to loop colostomy when colostomy is considered [17]. As a result, when the decision of surgical treatment in acute colon tumors is made; the general condition of the patient, the experience of the surgeon and hospital conditions should be taken into consideration. Early and late-term complications following colorectal cancer surgery are more frequent than those operated for benign indications. Probably cancer-specific deterioration of the immune system and fecal contamination have been blamed for these complications. In various studies performed, the morbidity and mortality rates of emergency colorectal surgery were up to 60% and 11%, respectively [18]. These rates have been given as 4-14% and 1-7% in elective colorectal cancer operations, respectively [19, 20]. In the present study, there was no statistically significant difference between the two groups in terms of Clavien-Dindo classification (2%, 21.0%, 17.8%, p = 0.577). Limitation of our study include the retrospective design and relatively small number of our series. In addition, some details of history and factors that may influence the outcome may not be completely documented. Lastly, higher proportion of patients who died and survived in our study group affected statistical results and we believe that the results may vary with larger series. Due to these restrictions, associations should be interpreted with caution.

## Conclusion

We found in our study that surgical interventions under urgent conditions and associated variable surgery durations in ASA 3 colon cancer patients do not have statistical significance with regard to short-term morbidity and mortality. Despite the screening programs applied in colorectal cancers, applications to emergency services and procedures performed under emergency conditions are still at high levels. Surgical operations, which have to be performed in patients with impaired metabolic status, carry major risks for patients, but their outcomes are also satisfactory for them. However, it has been reported that long-term outcomes of patients undergoing urgent surgery are worse than those of patients receiving elective surgery. Hence, we believe that it would be more appropriate to perform the surgery under elective conditions, if possible.

### What is known about this topic

- In the surgical treatment of obstructive colorectal cancers under emergency conditions, higher rates of morbidity and mortality are observed when compared with treatment under elective conditions;
- In this case, there are many treatment options such as ostomy, resection + ostomy or resection + anastomosis. In the literature, there are articles suggesting that only ostomy should be opened, but there are also reports indicating that resection may be also performed;
- There are different results related to the distribution of colorectal cancers according to gender in the literature.

### What this study adds

- In our study, no statistically significant difference was found between the study groups in terms of age and male/female distribution;
- When the decision of surgical treatment in acute colon tumors is made; the general condition of the patient, the experience of the surgeon and hospital conditions should be taken into consideration;
- We believe that it would be more appropriate to perform the surgery under elective conditions, if possible.
